# Micawberism

**Published:** 1899-05-13

**Authors:** 


### MICAWBERISM.

Professor Mayo Robson, in addressing the Nottingham Medico-Chirurgical Society on the Dangers of Delay, put into striking form a line of thought which must have drifted through the minds of most surgeons of large experience. There are, he says, certain minds that seem to adopt as their motto "Never do to-day what you can put off till to-morrow," and although in the ordinary affairs of life this may sometimes prove a more or less useful maxim, it is one which in medical and surgical matters may frequently mean the difference between life and death. Nor is this procrastination, which often leads to such great evils, to be laid, as some would have us lay it, at the door of the patient or his friends, for our clients will in nearly every instance be guided by us if they see that in our consultations we are united. The blame rather lies in the traditions of the past which we have not yet completely shaken off, in the reminiscences of the old days when an operation was a truly dreadful business, to be avoided if possible, and perhaps it lies a little in the *laissez faire* policy of the old school, for there are yet a good number of Micawbers in the world, and we know that they are sometimes encouraged in their methods by finding the unexpected turning up. To illustrate the serious consequences of delay Mr. Robson referred to a variety of cases.

Perhaps there is no more fertile cause of serious joint disease ending in deformity or death, than the delay which frequently occurs before absolute rest is advocated in the early stages of spinal or hip disease. From a large experience, he says, he is firmly convinced that if complete rest were insisted on in the early stages of these tuberculous joint cases, whether spine, hip, knee, elbow, or other joint, a complete and permanent recovery would occur in a very large number, probably in 95 per cent. of the cases. In nearly every case there is a history of injury or strain, may be months before, which is almost forgotten until recalled by the questions of the surgeon; this is followed by a little local injury near to or at an epiphysis, which under rest would entirely clear up, and which may lie dormant for a longer or shorter time until some other cause, such as deteriorated health, imperfect feeding, exposure to infection, further injury, or some unknown cause leads to rarefying osteitis with pain on movement. In this stage, again, complete rest would obviate further trouble in the great number of cases, but in the absence of proper treatment the inflamed area becomes the seat of tubercle, with its well-known sequelæ of abscess and destruction of bones and joints.

Cancer of the breast, again, is a good example of the evils of delay. "I think it is a good rule," he says, "to advocate the removal of every tumour of the breast at an early stage, which I feel sure is at present not the general rule, though advocated by many surgeons. Over and over again I have removed a scirrhus of the breast from a patient who has had a simple adenoma for years, the removal of which at an early stage would have been the simplest matter in the world. You never know when the epithelium in a simple adenoma may overstep the basement membrane and become a carcinoma. Therefore, delay in advocating surgical treatment is, to my mind, extremely unwise, especially as the simple operation is devoid of risk."

Nothing indeed is becoming more clearly established than that the early and complete removal of cancer is curative and not merely palliative, and this is shown equally well of cancer of the cervix uteri and of cancer of the rectum as in regard to cancer of the breast.

Perhaps of all striking cases of relief by well timed surgical interference none beat those of tuberculous peritonitis. "A few years ago," says Mr. Robson, "I was asked to see a young lady in this county who had been given up as beyond recovery by two able physicians who had seen her in consultation, and when I saw her lying in bed like a typhoid patient in the last stage of exhaustion, with sunken eyes, attenuated limbs, a hectic flush, extreme emaciation, a much enlarged abdomen, and dulness at the apex of the left lung, I almost felt inclined to say it was too late, but the simplest operation—an incision only large enough to admit a drainage tube—led to remarkable results, though the mesentery stood up stiff with tubercule, and tuberculous nodules were scattered all over the peritoneum; for she began to recover at once, and four months after she wrote to tell me she was so well as to be able to hunt and follow her usual country pursuits, and I have since heard of her in robust health." So much for a case operated on while the field for operation is open. But take the other side: "If the patient be not operated on and survive the stage of effusion we get matting of intestines and viscera generally, intestinal obstruction, caseation of glands, suppuration, and other troubles which frequently end fatally in one way or another." Moreover, the operation required to avert these evils is of the simplest. "An opening no bigger than to admit the finger and to allow of drainage is all that is required, and this can be done quite well under cocaine anæsthesia, the whole procedure not occupying more than a few minutes."

Other subjects from which the same lessons can be learned are appendicitis, gall stones, perforated gastric ulcer, perforated typhoid ulcer, dilatation of the stomach and stricture of the pylorus, intestinal obstruction, ovarian tumours, and even uterine tumours. Let us take ovarian tumours. "I have operated," he says, "some hundreds of times for large ovarian tumours, and the question has often crossed my mind, Why should these cases have been allowed to go on so long before being submitted to operation, when by bimanual examination they can be recognised in their infancy, and removed with almost absolute certainty of recovery before any complications have supervened"? It is the Micawberism, the waiting for something to turn up, which is the cause of so much delay, and it is far too often the delay which turns the small and simple operation, which might at the right time have been enough to cure, into the long and complicated one which is ultimately required.

